# Exploring the role of microRNAs as diagnostic and prognostic biomarkers in canine mammary tumors

**DOI:** 10.1007/s11357-024-01260-7

**Published:** 2024-07-02

**Authors:** Tiago Ferreira, Rui M. Gil da Costa, Francisca Dias, Adelina Gama, Vítor M. Gaspar, João F. Mano, Paula A. Oliveira, Rui Medeiros

**Affiliations:** 1 Centre for the Research and Technology of Agro-Environmental and Biological Sciences (CITAB), University of Trás-Os-Montes and Alto Douro (UTAD), 5000-801 Vila Real, Portugal; 2 Institute for Innovation, Capacity Building and Sustainability of Agri-Food Production (Inov4Agro), UTAD, 5000-801 Vila Real, Portugal; 3 Molecular Oncology and Viral Pathology Group, Research Center of IPO Porto (CI-IPOP)/RISE@CI-IPOP (Health Research Network), Portuguese Oncology Institute of Porto (IPO Porto)/Porto Comprehensive Cancer Center (Porto.CCC), 4200-072 Porto, Portugal; 4 Department of Chemistry, CICECO – Aveiro Institute of Materials, University of Aveiro, Campus Universitário de Santiago, 3810-193 Aveiro, Portugal; 5 Laboratory for Process Engineering, Environment, Biotechnology and Energy (LEPABE), Faculty of Engineering, University of Porto, Porto, Portugal; 6 Postgraduate Program in Adult Health (PPGSAD), Federal University of Maranhão (UFMA), São Luís, Brazil; 7 Animal and Veterinary Research Centre (CECAV), University of Trás-Os-Montes and Alto Douro (UTAD), 5000-801 Vila Real, Portugal; 8 Associate Laboratory for Animal and Veterinary Sciences (AL4AnimalS), University of Trás-Os-Montes and Alto Douro (UTAD), 5000-801 Vila Real, Portugal; 9 Faculty of Medicine of the University of Porto (FMUP), 4200-319 Porto, Portugal; 10 Research Department of the Portuguese League against Cancer—Regional Nucleus of the North (Liga Portuguesa Contra o Cancro—Núcleo Regional do Norte), 4200-177 Porto, Portugal; 11 Virology Service, Portuguese Institute of Oncology (IPO), 4200-072 Porto, Portugal; 12 Biomedical Research Center (CEBIMED), Faculty of Health Sciences of the Fernando Pessoa University, 4249-004 Porto, Portugal

**Keywords:** Cancer therapy, Dogs, Mammary tumors, miRNAs

## Abstract

Canine mammary tumors (CMTs) represent a significant health concern in dogs, with a high incidence among intact female dogs. CMTs are a promising comparative model for human breast cancer, due to sharing several pathophysiological features. Additionally, CMTs have a strong genetic correlation with their human counterpart, including the expression of microRNAs (miRNAs). MiRNAs are a class of non-coding RNAs that play important roles in post-translational regulation of gene expression, being implicated in carcinogenesis, tumor progression, and metastasis. Moreover, miRNAs hold promise as diagnostic, prognostic, and metastatic biomarkers. Understanding the molecular mechanisms underlying CMTs is crucial for improving diagnosis, prognosis, and monitoring of treatments. Herein, we provide a comprehensive overview of the current knowledge on miRNAs in CMTs, highlighting their roles in carcinogenesis and their potential as biomarkers. Additionally, we highlight the current limitations and critically discuss the overarching challenges in this field, emphasizing the need for future research to translate miRNA findings into veterinary clinical practice.

## Introduction

Dogs (*Canis lupus familiaris* Linnaeus, 1758) are popular companion animals in many countries [1]. In the last few years, the animal-owner relationship has become increasingly close, and advances in veterinary medicine have contributed to an increase in the life expectancy of animals. As in humans, one consequence of prolonged life expectancy is an increase in the amount of cancer cases [2]. Among the various types of cancer affecting domestic animals, canine mammary neoplasias are observed with notable frequency [3]. In fact, canine mammary tumors (CMTs) constitute the second most common group of neoplasms (after the skin) in intact female dogs, and although the majority are benign, they account for approximately 50% of all canine malignant lesions [4]. These lesions can occur in any breed and are age-associated, being more frequent in older animals. The average age at which mammary neoplasms appear is between 7 and 13 years [5–9]. This incidence is higher in geographical areas where ovariohysterectomy is not routinely performed or is performed at an older age [10]. Over 25% of unspayed female dogs will develop a mammary tumor at some point in their lifetime, with middle-aged to older dogs more susceptible [3]. The neoplastic lesions mainly appear in the caudal abdominal and inguinal mammary glands (around 65% of the cases), and multiple nodules are present in more than 50% of cases [5–9]. Typically, tumors are detected during a routine examination or reported by owners when they notice visible changes in the mammary gland. Surgery is the recommended elective treatment for CMTs, but adjuvant therapies can also be used [11]. Regrettably, adjuvant therapies are expensive and there is no guarantee of results, leading owners to refuse them [12]. The most common benign lesions are mixed tumors, followed by complex adenomas and simple adenomas. The most prevalent malignant tumors are simple tubulopapillary and complex carcinomas [13]. Like human breast cancer, canine mammary carcinomas are categorized into three histological grades (grade I, II or III) according to tubule formation, nuclear pleomorphism and mitotic index [14]. As in humans, microRNAs (also known as miRNAs or miRs) have been shown to play a critical role in the development and progression of mammary tumors in dogs [15–17]. Studies have identified miRNAs that are differentially expressed in malignant mammary tumors relative to normal mammary tissue and are related to their pathogenesis, namely by modulating cell proliferation, histological type and grade, and metastasis [18–21]. This differential expression can also be used to predict the course of the disease [18, 19, 22], suggesting that miRNAs may be potential diagnostic and prognostic biomarkers in this type of cancer [16, 23, 24].

### MicroRNAs and biogenesis

MiRNAs are small endogenous non-coding RNAs involved in post-translational repression of gene expression [25]. MiRNAs mainly bind to specific sites in the 3' untranslated region (UTR) of target messenger RNA (mRNA) molecules, leading to the inhibition of translation or degradation of the mRNA. Consequently, there is a suppression of protein production from the target gene [25]. Thousands of miRNAs have been found in different organisms, including eukaryotic, prokaryotic and virus, that perform distinct functions at different times. These molecules are phylogenetically conserved in several species, including humans and dogs, and it is estimated that around 30% of the mammalian protein-coding genome is regulated by miRNAs [24, 26, 27].

MiRNAs play a significant role in many biological processes, such as cell cycle, proliferation, differentiation and apoptosis [28–31]. MiRNAs are found in all tissues and body fluids, including blood, serum, plasma, urine, saliva, semen, pleural and ascitic effusion [32]. In pathological conditions, namely in human cancers, miRNAs are one of the most studied subclasses of non-coding RNAs (ncRNAs) due to their significant role in cancer development [33]. In dogs, miRNAs also play an important role in carcinogenesis; however, there is still limited research in this area [34].

In terms of nomenclature, mature miRNA sequences are denoted by “miR”, while their stem-loop pre-miRNA molecules are denoted by ‘mir’ within miRNA names [35, 36]. This nomenclature is applicable to all miRNAs, with the exception of the “let”-family, which includes some of the first miRNAs discovered. Three- or four-letter prefixes are added to the term “miR” to indicate the organism from which the miRNA was identified, e.g., hsa for *Homo sapiens* (hsa-miR) or cfa for *Canis lupus familiaris* (cfa-miR). The number is added afterwards to indicate the sequential order of discovery (e.g. cfa-miR-21) [36]. Identical mature miRNA sequences transcribed from distinct precursor sequences and genes are assigned number suffixed identities such as cfa-miR-219–1 and cfa-miR-219–2 [37]. Mature miRNAs with only one or two nucleotide sequence differences are differentiated by lettered suffixes, such as cfa-miR-15a and cfa-miR-15b [37].

**Fig. 1:**
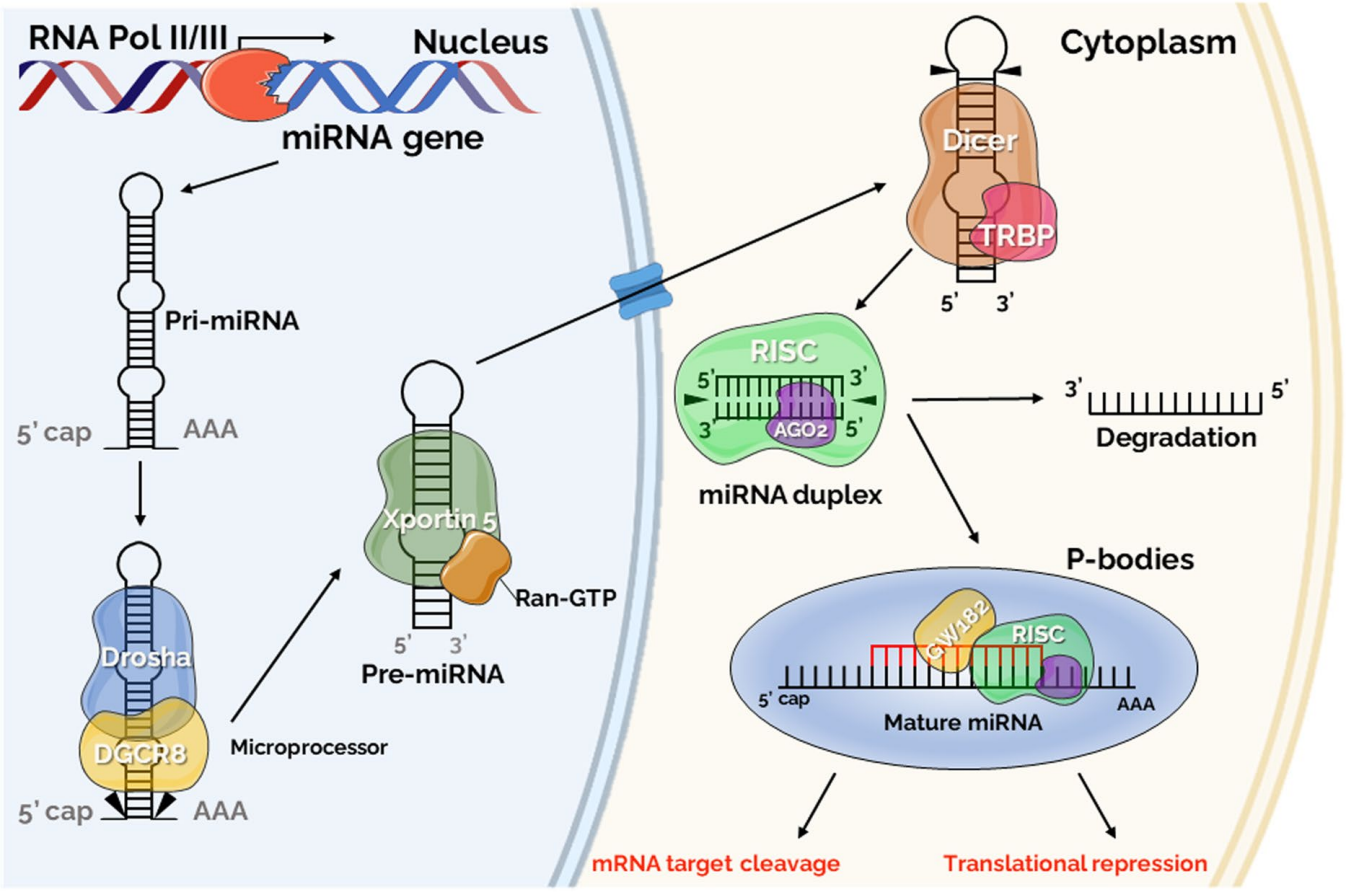
The canonical miRNA biogenesis pathway. Ago2: Argonaute; DGCR8: DiGeorge Syndrome Critical Region 8; P-body: Processing bodies; Pre-miRNA: Precursor-miRNAs; Pri-miRNA: Primary miRNA; Ran-GTP: RAS-related nuclear protein-guanosine-5’-triphosphate-ase; RISC: RNA-induced silencing complex; TRBP: Transactivation response RNA binding protein; XPO5: Exportin-5. (Parts of the figure were drawn by using pictures from Servier Medical Art, provided by Servier, licensed under a Creative Commons Attribution 3.0 Unported license)

The production of miRNAs involves a series of steps, including transcription, processing, and transport, to ensure proper maturation and function within the cell (Fig. 1). In the canonical pathway, primary miRNA (pri-miRNA) transcription occurs in the nucleus, forming precursor miRNA (pre-miRNA) through cleavage by the microprocessor complex, constituted by Drosha and DiGeorge Syndrome Critical Region 8 (DGCR8) protein [38–42]. Subsequently, the pre-miRNA is transported to the cytoplasm by an exportin-5 and RAS-related nuclear protein-guanosine-5’-triphosphate-ase (Ran-GTP) complex, where it is processed by the Dicer to form a mature miRNA duplex. [38, 43, 44]. The miRNA-Dicer complex joins the RNA-induced silencing complex (RISC) with Argonaute 2 (Ago2), protein kinase RNA activator (PACT) and trinucleotide repeat-containing gene 6A (TNRC6A) [45, 46]. Ago2 interacts with miRNA and one strand (the passenger strand) is degraded. The guide strand, which remains bound to Ago2, pairs with GW182 family members and together induces degradation or translational inhibition of the target mRNA [37]. This process occurs in cytoplasmic processing bodies (P-bodies), which contain a high concentration of enzymes and factors needed for translational repression [47]. The mode of action of the mature miRNA to inhibit or degrade the target mRNA depends on its complementarity. When miRNAs attach to their target mRNA with complete or near-perfect complementarity, they cause cleavage of the target mRNA. When miRNAs bind to their target mRNA with imperfect complementarity, translational deletion occurs [48]. Different miRNAs are able to target the same mRNA, while a single miRNA acts on many targets, thus regulating multiple pathways and affecting the expression of many genes [49]. To further add to this complexity, miRNAs can interact directly with other miRNAs, regulating their activity [50, 51].

MiRNA expression can be dysregulated as a result of chromosomal abnormalities, mutations, single nucleotide polymorphisms, epigenetic alterations, errors in miRNA biogenesis, and changes in transcription factors [52]. In humans and dogs, several diseases have been associated with dysregulated miRNA expression, including breast/mammary gland cancer [18, 19, 22, 53]. Depending on how their target genes operate, miRNAs can either act as tumor suppressors or oncogenes (oncomiRs) [54]. OncomiRs have been reported to be overexpressed in cancer and are known to downregulate tumor suppressor genes. Conversely, tumor suppressor miRNAs are underexpressed in cancer and are responsible for downregulation of oncogenes [55].

One of the best known and most studied oncomiRs is miR-21. This miRNA is overexpressed in several types of cancer, particularly breast cancer [56–59]. It is associated with advanced clinical stage, lymph node metastasis and a poor prognosis in primary breast cancer [58]. In addition, a higher miR-21 expression is associated with positive estrogen and progesterone receptor status [59]. Moreover, this miRNA targets several tumor suppressor genes in breast cancer, such as metalloproteinases-3 (TIMP3), tropomyosin-1 (TPM1), TP53, programmed cell death protein 4 (PDCD4), phosphatase and tensin homolog (PTEN), and maspin, repressing their functions [60–62]. Other examples of oncomiRs are miR-155, miR-10b and miR-221/222 which are well-known in the pathogenesis and treatment of breast cancer [63]. An example of tumor suppressor miRNAs is miR-125 family [64]. miR-125b is located on chromosome 11q24 and chromosome 21q21, which are frequently deleted in breast cancer, indicating a functional loss of miR-125b in this type of tumor [65]. A study indicated that miR-125b expression was decreased in invasive breast cancer tissues and that hypermethylation of miR-125b promoter was partly responsible for the reduction in miR-125b expression. This miRNA targets the *EST1* gene, lower expression of miRNA-125 and high levels of *EST1* predict poor survival in breast cancer patients [66]. This tumor suppressor miRNA has other targets such as ERBB2, ERBB3 and cytochrome P450 family 24 subfamily A member 1 (CYP24) [64]. Let-7, miR-200, miR-205 and miR-206 families are other tumor suppressor miRNAs studied in breast cancer [64]. Therefore, the present review aims to systematize the information available of miRNAs in CMTS, bringing an update on this field, their associations with carcinogenesis and their potential as biomarkers.

## Evidence acquisition

A bibliographic search was carried out on PubMed and Web of Science, using the search terms “((microRNA OR miRNA) AND (canine mammary tumors OR canine mammary carcinoma))”. This search resulted in 60 articles (16 from Web of Science and 44 from PubMed) published between 2008 and 2023. After removing duplicates (n = 15), of the remaining articles (n = 45), 16 were excluded based on the following exclusion criteria, which included: review articles, meta-analyses, retracted articles, and studies on types of cancer other than mammary or species than dogs, thus resulting in 29 original articles. Additionally, inclusion criteria were also applied based on the following: (1) in vitro and in vivo studies, and studies with female dogs; (2) studies that focused on miRNAs. Following the application of these criteria, 9 articles were excluded. Therefore, a total of 20 articles served as the basis for this review.

## Evidence synthesis

### MicroRNAs in canine mammary tumors

**Fig. 2:**
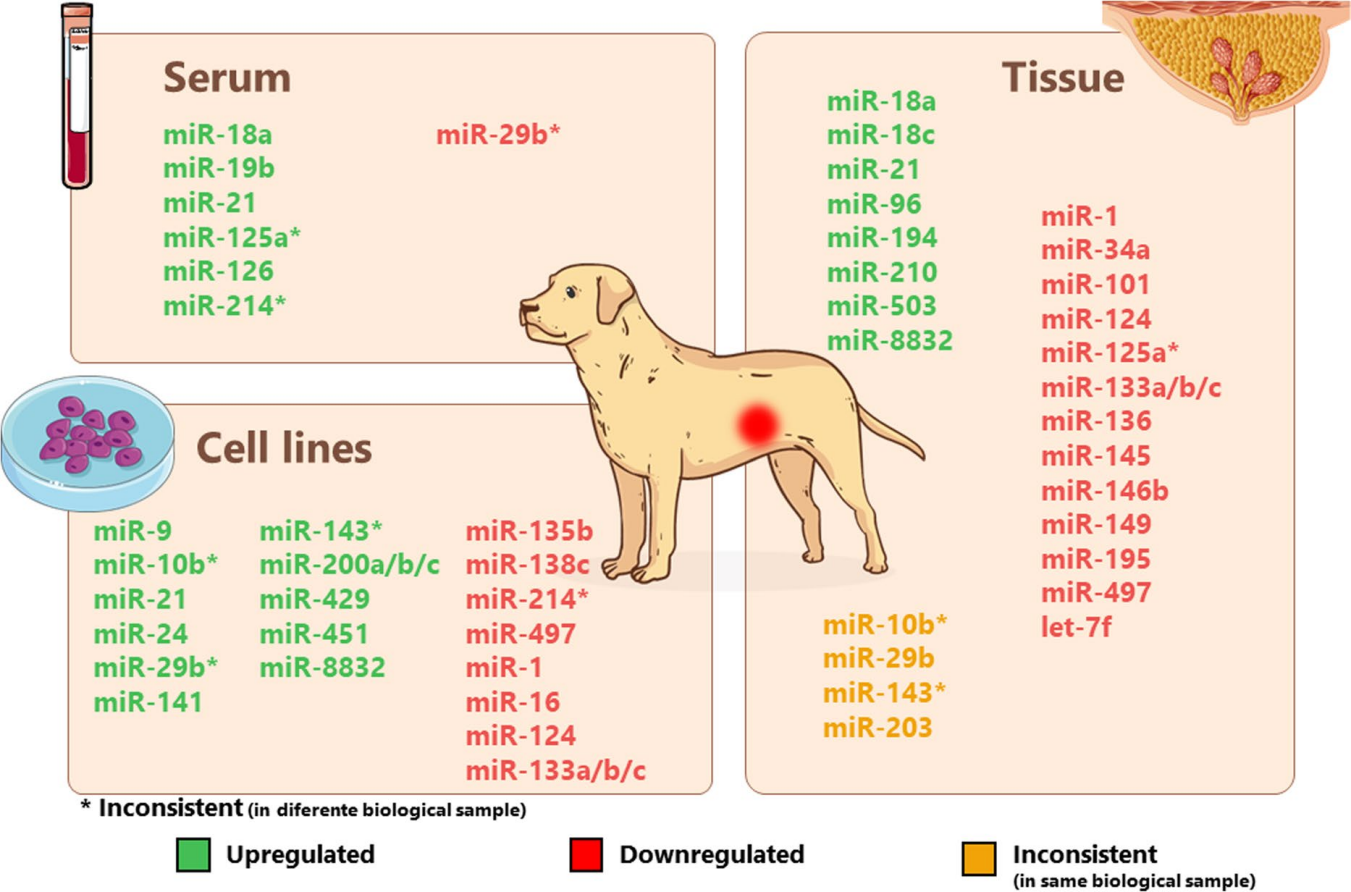
Expression of miRNAs in CMTs. Upregulated miRNAs are marked in green and downregulated ones in red. The miRNAs in yellow are those that were found to be inconsistent in the same biological sample. * Represent the same miRNAs with controversial results between different biological samples. (Parts of the figure were drawn by using pictures from Servier Medical Art, provided by Servier, licensed under a Creative Commons Attribution 3.0 Unported license)

MiRNAs have been shown to play a vital role in the development and progression of mammary tumors in dogs (Fig. 2) [21, 22, 67]. In contrast to the research on miRNAs related to human breast cancer, research into canine mammary cancer miRNAs is limited. Nevertheless, the mature miRNAs share a high degree of homology between the two species which means that various human miRNA assays can be also used to analyze the expression of canine miRNAs [16, 51]. The selected articles analyzed miRNAs from sources such as canine mammary cancer cell lines [67–73], canine mammary tumor tissues [18–21, 71, 73–77], and canine blood serum [22, 78–81], through different techniques. Table 1 summarizes potential use of miRNAs in the clinic as biomarkers for diagnosis, prognosis, prediction of metastasis and/or response to therapy.

**Table 1:** Expression of miRNAs associated with CMTs and their potential application in clinical practice

microRNA	Sample	Cohort size	Detection method	Expression level	Biomarker	References
miR-1	Cell line	*n* = 3	RT-qPCR	Down	Diagnostic	[67]
	Tissue	*n* = 92	RT-qPCR	Down		[77]
miR-9	Cell line	*n* = 3	RT-qPCR	Up	Diagnostic	[67]
miR-10b	Tissue	*n* = 146	Microarray	Down	Diagnostic/metastatic	[22]
	Tissue	*n* = 16	RT-qPCR	Up		[76]
	Cell line	*n* = 1	Microarray	Up		[69]
miR-16	Cell line	*n* = 1	RT-qPCR	Down	Diagnostic	[70]
miR-18a	Exosomes	*n* = 5	NGS	Up	Diagnostic/prognostic	[68]
	Serum	*n* = 10	NGS	Up		[78]
	Tissue	*n* = 26	RT-qPCR	Up		[20]
	Tissue	*n* = 26	RT-qPCR	Up		[19]
miR-18b	Tissue	*n* = 26	RT-qPCR	Up	Diagnostic/prognostic	[20]
	Tissue	*n* = 26	RT-qPCR	Up		[19]
miR-19a	Exosomes	*n* = 5	NGS	Up	Diagnostic	[68]
miR-19b	Serum	*n* = 10	NGS	Up	Diagnostic	[78]
miR-21	Cell line	*n* = 1	RT-qPCR	Up	Diagnostic/prognostic/metastatic	[70]
	Serum	*n* = 40	RT-qPCR	Up		[80]
	Serum	*n* = 10	RT-qPCR	Up		[81]
	Tissue	*n* = 16	RT-qPCR	Up		[18]
	Tissue	*n* = 16	RT-qPCR	Up		[76]
	Tissue	*n* = 146	Microarray	Up		[22]
	Tissue	*n* = 26	RT-qPCR	Up		[20]
	Tissue	*n* = 26	RT-qPCR	Up		[19]
miR-24	Cell line	*n* = 1	RT-qPCR	Up	Diagnostic	[70]
miR-29b	Tissue	*n* = 16	RT-qPCR	Up	Diagnostic/prognostic/metastatic	[18]
	Cell line	*n* = 1	Microarray	Up		[69]
	Cell line	*n* = 1	RT-qPCR	Up		[70]
	Tissue	*n* = 30	RT-qPCR	Down		[21]
	Tissue	*n* = 146	Microarray	Down		[22]
	Serum	*n* = 40	RT-qPCR	Down		[80]
miR-34a	Tissue	*n* = 16	RT-qPCR	Down	Diagnostic	[76]
miR-96	Tissue	*n* = 6	RT-qPCR	Up	Diagnostic	[74]
miR-101	Tissue	*n* = 30	RT-qPCR	Down	Diagnostic/prognostic/metastatic	[21]
	Tissue	*n* = 146	Microarray	Down		[22]
miR-124	Tissue	*n* = 20	RT-qPCR	Down	Diagnostic/therapeutic	[71]
	Cell line	*n* = 1	RT-qPCR	Down		
miR-125a	Serum	*n* = 10	NGS	Up	Diagnostic/prognostic/metastatic	[78]
	Tissue	*n* = 30	RT-qPCR	Down		[21]
	Tissue	*n* = 146	Microarray	Down		[22]
miR-125b	Tissue	*n* = 146	Microarray	Down	Metastatic	[22]
miR-126	Serum	*n* = 7	RT-qPCR	Up	Diagnosis/prognosis	[79]
miR-133a	Tissue	*n* = 92	RT-qPCR	Down	Diagnostic	[77]
	Cell line	*n* = 3	RT-qPCR	Down		[67]
miR-133b	Tissue	*n* = 92	RT-qPCR	Down	Diagnostic	[77]
	Cell line	*n* = 3	RT-qPCR	Down		[67]
miR-133c	Tissue	*n* = 92	RT-qPCR	Down	Diagnostic	[77]
	Cell line	*n* = 3	RT-qPCR	Down		[67]
miR-135b	Cell line	*n* = 3	Microarray	Down	Diagnostic	[72]
miR-136	Tissue	*n* = 146	Microarray	Down	Metastatic	[22]
miR-138a	Cell line	*n* = 1	Microarray	Down	Diagnostic	[69]
miR-141	Cell line	*n* = 3	RT-qPCR	Up	Diagnostic/prognostic	[67]
miR-143	Tissue	*n* = 146	Microarray	Down	Diagnostic/metastatic	[22]
	Tissue	*n* = 30	RT-qPCR	Down (in carcinoma)	Diagnosis/prognostic/metastatic	[21]
	Tissue	*n* = 30	RT-qPCR	Up (in metastasis)		
	Cell line	*n* = 1	Microarray	Up	Diagnostic	[69]
miR-145	Tissue	*n* = 30	RT-qPCR	Down	Diagnostic/prognostic/metastatic	[21]
	Tissue	*n* = 146	Microarray	Down		[22]
miR-146b	Tissue	*n* = 26	RT-qPCR	Down	Diagnostic/prognostic	[20]
miR-149	Tissue	*n* = 6	RT-qPCR	Down	Diagnostic	[74]
miR-181a	Exosomes	*n* = 5	NGS	Up	Diagnostic	[68]
miR-194	Tissue	*n* = 30	RT-qPCR	Up	Diagnostic	[21]
miR-195	Tissue	*n* = 30	RT-qPCR	Down	Diagnostic	[73]
miR-200a/b	Cell line	*n* = 3	RT-qPCR	Up	Diagnostic	[67]
miR-200c	Cell line	*n* = 1	RT-qPCR	Up	Diagnostic/prognostic	[70]
	Cell line	*n* = 3	RT-qPCR	Up		[67]
miR-203	Tissue	*n* = 146	Microarray	Down	Diagnostic/metastatic	[22]
	Tissue	*n* = 30	RT-qPCR	Up		[21]
miR-210	Tissue	*n* = 30	RT-qPCR	Up	Diagnostic/prognostic/metastatic	[21]
	Tissue	*n* = 146	Microarray	Up		[22]
miR-214	Serum	*n* = 7	RT-qPCR	Up	Diagnostic/prognostic	[79]
	Cell line	*n* = 3	RT-qPCR	Down		[67]
miR-429	Cell line	*n* = 3	RT-qPCR	Up	Diagnostic	[67]
miR-451	Cell line	*n* = 3	Microarray	Up	Diagnostic	[72]
miR-497	Tissue	*n* = 30	RT-qPCR	Down	Diagnostic/therapeutic	[73]
	Cell line	*n* = 2	RT-qPCR	Down		[73]
miR-503	Tissue	*n* = 20	NGS	Up	Diagnostic/prognostic	[75]
miR-8832	Tissue	*n* = 6	RT-qPCR	Up	Diagnostic	[74]
let-7f	Tissue	*n* = 146	Microarray	Down	Metastatic	[22]

NGS: next-generation sequencing; RT-qPCR: reverse transcription-quantitative polymerase chain reaction

Most of the studies used reverse transcription-quantitative polymerase chain reaction (RT-qPCR) as their preferred method for miRNA analysis, but there were also some studies that used microarray or next-generation sequencing (NGS).

The first study of miRNA expression in CMTs was published in 2008 and miRNAs relevant to human breast cancer were investigated by RT-qPCR [18]. The study analyzed six malignant CMTs and ten normal canine mammary tissues (average of 8 years with a range 4–11) and described the expression patterns of ten miRNAs: miR-15a, miR-16, miR-17-5p, miR-21, miR-29b, miR-125b, miR-145, miR-155, miR-181b and let-7f. The authors concluded that the miRNAs associated with human breast cancer follow the same expression pattern in dogs, except for miR-145. Furthermore, this study observed that miRNA-29b and miRNA-21 were statistically significant upregulated in tumor samples relative to normal tissue. The authors also compared the samples based on two histological types. They observed that miR-181b, miR-21, miR-29b and let-7f were upregulated in tubulopapillary carcinomas and that miR-15a and miR-16b were downregulated in ductal carcinomas. Their study suggests that miR-15a and miR-16b may be involved in tumor differentiation [18].

One study analyzed the expression of miRNA-21, miRNA-10b and miRNA-34a in benign and malignant tumors by RT-qPCR using thirteen tumor and three healthy tissue samples. The authors observed increased expression of miRNA-21 and miRNA-10b and decreased expression of miRNA-34a in benign and malignant tumors compared to normal tissue [76].

Researchers investigated by RT-qPCR the expression of 16 miRNAs (miR-136, miR-143, let-7f, miR-29b, miR-145, miR-9, miR-10b, miR-203, miR-125b, miR-15a, miR-16, miR-21, miR-101, miR-210, miR-194 and miR-125a) in 20 tissue samples of different tumors including adenomas, non-metastasizing and metastasizing mammary carcinomas as well as lymph node metastasis. Tumors at different stages of malignancy varied considerably from normal glands in the expression of seven miRNAs – miR-203, miR-210, miR-143, miR-21, miR-194, miR-145 and miR-101. miR-210 expression was increased in all neoplastic tissues compared to normal tissue. Metastatic tissues differed from primary or non-metastasizing tumors in the expression of mir-29b, miR-101, mir-145, miR-143 (downregulated) and miR-125a (upregulated). However, increased miR-143 expression was observed in the metastatic group compared to normal tissue. Comparison of miRNA expression in primary tumors of different malignancies (i.e., adenoma, non-metastatic carcinoma, and metastatic carcinoma) revealed no significant differences, except for significant downregulation of miR-125a in metastatic carcinomas compared to adenomas [21]. miR-29b has been described as downregulated in cancer-associated fibroblasts, and this underexpression plays a significant role in tumor stroma through the activation of p38-STAT1 in human breast cancer cells [82]. However, another study observed that higher expression of miR-29b was associated with a lower survival rate in patients with breast cancer [83]. In fact, this miRNA has a controversial role, since it acts as a tumor suppressor or oncomiRs in breast cancer [84]. A recent study observed that miR-101 was downregulated in breast cancer samples and cell lines and its overexpression inhibited the malignant behavior of human breast cancer cells. The authors concluded that miR-101 plays a role as tumor suppressor, targeting the sex-determining region Y-box 2 (SOX2). This upregulated oncogene was associated with lymph node metastasis and pathological grade in breast cancer [85].

A recent study evaluated 277 miRNA expression profiles using RT-qPCR from three canine mammary cancer cell lines — CMT12, CMT27, and CMT2 — derived from female dogs of different breeds with spontaneous mammary carcinomas. Of the 277 miRNAs analyzed, 151 were differentially up- and/or downregulated in at least one cell line when compared with normal canine mammary epithelial cells. Forty-five miRNAs were increased and 24 were decreased in all three cell lines. The miR-1, miR-133a/b/c and miR-214 were highly downregulated in three canine mammary cancer cell lines, whereas miR-203, miR-9, miR-429, or miR-200a/b were found highly upregulated in all canine mammary cancer cell lines. In contrast, miR-141 and miR-200c were observed to be highly upregulated in CMT12 and CMT27 cell lines and were identified as highly downregulated in the CMT28 cell line. In this work, it was also experimentally proven that miR-141 targets the 3’-UTR of the tumor suppressor p16^INK4^ (inhibitor of CDK4) through a direct link between overexpression of miR-141 and the target mRNA p16^INK4A^ in the cell lines CMT12 and CMT27, emphasizing that miR-141 is a relevant oncomiR. In addition, the authors correlated the data with human breast cancer and recognized some similarly altered miRNAs, including miR-21, miR-155, miR-9 (all upregulated), miR-34a, miR-143/145, and miR-31 (all downregulated) [67].

Another study assessed the miRNA profile in a canine mammary carcinoma cell line obtained from the pleural effusion of a 10-year-old mixed-breed dog. The miRNA profile was determined by microarray from the carcinoma cell line, and it was compared with normal canine mammary tissue. The authors observed 291 altered miRNAs, with the expression of miRNA-143 and miRNA-138a showing a high increase and decrease, respectively. The authors also suggested that the observed upregulation of miR-10b, miR-29b and mi-143 is associated with CMTs [69].

Using a microarray, a research team analyzed miRNA profiles in canine mammary carcinoma stem-like cells obtained by cell sorting from three canine mammary carcinoma cell lines (i.e., CMT-U27, CMT-309 and P114). When comparing cancer stem-like cells to well-differentiated tumor cells, the researchers observed 24 downregulated and 9 upregulated miRNAs. In that study, miRNA-451 was found to be the most significantly upregulated and miRNA-135b was found to be the most significantly downregulated [72]. miRNA-451 have been described as oncomiRs that influences the progression of tumorigenesis. In fact, numerous mRNAs have been identified as miR-451 targets, covering a wide range of biological signaling pathways, such as cell proliferation (i.e., CDKN2D and MAP3K1), apoptosis (i.e., IKK-β), invasion, (i.e., ATF2) migration (i.e., c-Myc), epithelial-mesenchymal transition (EMT; i.e., c-Myc) and angiogenesis (i.e., MIF) [86]. Furthermore, overexpression of miRNA-451 was shown to have chemoresistance effects, as it increased human breast cancer cell resistance to paclitaxel through negative regulation of tyrosine3-monooxygenase/tryptophan5-monooxygenase activation protein zeta (YWHAZ) [87]. The role of miRNA-135b as a tumor suppressor has been described in human breast cancer cell lines and murine models. A recent study suggests that miR-135b suppresses cell proliferation, migration, invasion and EMT in the MDA-MB-468 and MCF-7 cell lines by inhibiting the Wnt/β-catenin pathway [88].

Another study established and characterized a canine mammary cancer cell line (FR37-CMT) derived from a grade II mammary tumor. The authors evaluated the expression of seven miRNAs by RT-qPCR—miR-16, miR-21, miR-24, miR-29b, miR-124, miR-155 and miR-200c. The results showed that miR-16, miR-21, miR-24, miR-29b and miR-200c were downregulated in relation to normal tissue. Interestingly, miR-21, miR-29b and miR-200c were also significantly under-expressed compared to the original tumor [70].

In a study by Bulkowska and colleagues, the profiles of 317 miRNAs were determined in 146 female dogs with CMT and 25 control samples, by using microarray. According to the study, a total of 124 miRNAs were found to differ significantly (including 98 miRNAs downregulated and 26 miRNAs upregulated in the metastatic group compared to the non-metastatic group), indicating that miRNAs may be useful as biomarkers of metastasis. miR-29b, miR-101, miR-143, miR-145 and miR-125a were some of the miRNAs found downregulated in a metastatic group in relation to the benign group. In addition, miR-210 was overexpressed in tumors compared to the control group, while miR-21 was upregulated in non-metastasizing tumors in comparison with the control group. Furthermore, miR-203 was downregulated in benign tumors compared to control group. Finally, the authors noted that miR-10b, miR-125b, miR-136 and let-7f expression levels decreased from normal mammary tissue, through benign tumors and non-metastatic malignant tumors, to metastatic tumors [22].

In 2018, the first study reporting the release of exosome-derived miRNA by canine mammary cells was conducted. For that, researchers isolated exosomes from three normal canine mammary epithelial cell cultures derived dogs without mammary pathology (i.e., CMEC1, CMEC2, CMEC3) and five canine mammary tumor cell lines with histopathology diagnoses the mammary carcinoma (i.e., CMT12, CMT27, CMT28, CMT47, CMT119) derived from different breeds. By using NGS to analyze exosomal miRNAs (exomiRs), a total of 145 miRNAs were found differentially expressed (i.e., 118 overexpressed and 27 underexpressed) compared to control cells. Three miRNAs (miR-18a, miR-19a, and miR-181a) were selected for validation by RT-qPCR. These miRNAs were also revealed to be upregulated. This study suggests that the identification of miRNAs secreted by exosomes that can be eliminated in biofluids such as blood and urine may allow their use as non-invasive biomarker[68].

The researchers evaluated six miRNAs (miR-141, miR-8893, miR-8908e, miR-96, miR-8832, miR-8852) in only six CMTs from female dogs aged between 3 and 14 years (average 10 years) by RT-qPCR. The results demonstrated the existence of a novel miRNA associated with CMTs, miR-8832, which was overexpressed compared to normal tissue. Furthermore, miR-96 and miR-149 were observed upregulated and downregulated, respectively [74].

Using RT-qPCR, a recent study analyzed miRNAs expression from ten normal, six tumor-adjacent and seventy-six tumor-bearing mammary gland tissue. This study observed that miR-1-3p, miR-133a-3p, miR-133b-3p, and miR-133c-3p showed downregulated in tumor samples compared to normal and tumor-adjacent tissue [77].

More recently, an interesting study evaluated the expression of seven miRNAs (i.e., miR-18a, miR-18b, miR-22, miR-124, miR-145, miR-21, miR-146b) in twenty-six formalin-fixed, paraffin-embedded mammary samples by RT-qPCR. These samples included 22 CMTs (7 benign; 15 malignant) and 4 control samples (3 normal mammary glands and 1 case of lobular hyperplasia) with an average age of 10 years (range 4–12 years). miR-18a, miR-18b and miR-21 were upregulated in malignant tumors relative to control tissues, while miR-146b was downregulated in benign and malignant mammary tumors relative to control samples. In addition, miR-146b was also observed with upregulation in grade II malignant tumors compared to grade I and III tumors and benign tumors [20]. miR-18a and miR-18b target *ESR1* mRNA that encodes for the estrogen receptor alpha (ERα). In a second study, the same researchers observed that the upregulation of these miRNAs was inversely associated with the expression of *ESR1* target mRNA and progressive loss of ERα immunoexpression and that these patterns were associated with malignant tumors. In addition, using the ki-67 index, the authors observed that miR-18a and miR-18b overexpression were associated with increased tumor cell proliferation [19]. In fact, loss of ERα immunoexpression and decrease of *ESR1* mRNA levels associated with an increase in the stage and grade of CMTs have been previously described [89–92]. A recent study analyzed twelve benign and eight malignant samples by NGS and concluded that miR-503 was expressed differently according to malignancy, and could be used as a potential biomarker in the assessment of malignant CMTs [75]. In sum, these miRNAs may be considered potential candidates for diagnostic and prognostic biomarkers.

### Circulating miRNAs

In addition to CMT samples analysis, circulating miRNAs can be studied in body fluids, such as blood, as a non-invasive method. We found five studies that reported miRNAs differentially expressed in the serum of dogs with CMTs, and some of these miRNAs may also serve as diagnostic and prognostic markers [22, 78–81].

The first study to analyze circulating miRNAs was conducted in 2017, using blood samples of 50 female dogs with mammary tumors and 12 healthy female dogs. Blood samples were used to validate the results obtained by microarray by analyzing the three miRNAs most downregulated in tumor samples of the metastatic group (miR-144, miR-32 and miR-374a) by RT-qPCR. These miRNAs revealed no significant differences in blood samples, suggesting no correlation between the miRNA profiles recorded in tumor and blood samples [22].

In addition, miR-214 and miR-126 were also upregulated in the serum of seven female dogs with CMTs using RT-qPCR [79]. A study using serum samples from ten female dogs with CMTs (10.5 years) and ten healthy female dogs (five intact and five neutered; 3 years) assessed the expression of various biomarkers. Using NGS and digital droplet PCR, the authors analyzed miR-18a, miR-19b, miR-29b, miR-34c, miR-122, miR-125a, and miR-181a and compared them with clinicopathological features such as grade, metastasis and survival. miR-19b and miR-125a were significantly overexpressed in CMTs compared to healthy samples. In addition, miR-18a was significantly increased in female dogs with stage III tumors compared with stage I/II tumors. The same miRNA was observed when lymphatic invasion was detected on histological analysis. This study concluded that serum miR-19b and miR-125a could be used as diagnostic biomarkers and that miR-18a can be used as a prognostic marker as it can be a predictive biomarker of metastasis [78]. Overexpression of miR-19b has been demonstrated to promote human breast cancer cell migration and metastasis by downregulating the myosin regulatory light chain interacting protein (MYLIP), involved in regulating cell movement and migration. Furthermore, this miRNA also affects the expression levels of cell adhesion molecules (including E-Cadherin, ICAM-1 and Integrin β1) [93]. Interestingly, in human breast cancer cell lines, miR-125a act as a tumor suppressor and is underexpressed, interfering with the expression of ERBB2, ERBB3 and HUR (an RNA binding protein), promoting antiproliferative effects [94, 95].

A research work analyzed serum samples from 60 female dogs, 20 with benign tumors, 20 with malignant tumors and 20 as control group, by RT-qPCR. In this study, the group with tumors had an average age of 8.25 and the control group had an average of 6.75. The authors observed that miR-21 expression differed significantly between healthy/control, benign mammary tumors and malignant mammary tumors, with higher overexpression in malignant tumors. The authors also observed downregulation of miR-29b of both benign and malignant tumors relative to control. However, they did not observe differences between tumor samples. This study suggests that miR-21 overexpression can be used as a non-invasive prognostic biomarker for the early detection of CMTs and that miR-29b can increase the accuracy of CMT detection when measured in conjunction with miR-21 [80].

Another study noted that miR-21 is a promising non-invasive biomarker, as serum levels of miR-21 were higher in CMTs than other serum tumor markers such as CA15-3 (cancer antigen 15–3). For that, in this work were included ten serum samples from animals with mammary tumors and 7 control samples from animals without mammary tumors (average of age 9 years, range 7–15 years) and analyzed by RT-qPCR. The authors also reported that miR-21 was overexpressed in vimentin-positive tumors, suggesting a mesenchymal origin of the tumor cells or occurrence of EMT [81].

Figure 2 summarizes the distinct expression patterns of miRNAs in dogs with CMTs across various biological samples, including serum, mammary tissue, and cancer cell lines.

## Challenges and future perspectives

There is a need to increase veterinary research in the mammary cancer area as studies are still scarce [16]. However, looking at the existing studies, there are some challenges to overcome. Those challenges include the need for standardization of the samples and the technique used for analysis. The results of the different sample types were sometimes contradictory [22]. The sample size and heterogeneity must be greater, as well as the availability of high-quality clinical samples. Integration of miRNA data with other molecular and clinical features is also needed. Large-scale studies and multicenter collaboration can overcome this challenge.

At the same time, the heterogeneity of miRNAs is a complex aspect that raises the need for personalized approaches that can help understand the mechanisms underlying this heterogeneity, thus improving therapeutic results. To this aim, bioengineered i*n vitro* models represent a valuable tool to help researchers study the specific function of individual or groups of miRNAs in a pre-clinical context. In this sense, personalized 3D in vitro models such as spheroids and organoids engineered from animal’s own cells and animal tissue-specific decellularized extracellular matrix are particularly relevant to study fundamental aspects of miRNAs in cancer [5]. This approach holds an important advantage since it mimics the tumor microenvironment and tissue-tissue interactions, thereby allowing for a more robust identification of miRNAs. In fact, with these biomimetic models it may be possible to obtain an animal-specific miRNA profile with results that are more similar to the in vivo scenario than 2D models [96, 97]. In summary, this approach could provide more precise insights on how miRNAs play complex regulatory roles in cellular systems and responds to different pathological and therapeutics conditions.

On the other hand, aging and its associated consequences must also be considered in this field [98]. Although there are few studies on the synergistic role of miRNA in aging and cancer in human medicine, it is known that there is an intrinsic relationship between them [99–101]. Dysregulation of miRNAs has been associated with age-related disease, particularly cancer, contributing to an increased risk of cancer development [102, 103]. A study observed that the miRNA profile is distinct among young women with breast cancer and older women with breast cancer [104]. In CMTs, to the best of our knowledge, no studies have been published analyzing microRNAs by age group. In fact, most studies do not mention age, with only one mentioning the age of controls and cases samples separately [80], making it unclear whether age influences the expression of microRNAs related to CMTs. Future studies should investigate animal groups by age to determine if age-related changes interfere with the treatment outcomes. Furthermore, dividing the groups according to breed is necessary, as some breeds live longer than others [105]. Large breeds have been diagnosed with cancer earlier than small and medium dogs [106]. Additionally, because of the genetic heterogeneity of breeds, it is important to analyze them separately [107, 108]. For example, purebred dogs were diagnosed at a younger age than mixed dogs [106]. In humans, divergent expression of miRNAs was observed in women with breast cancer across different ethnic groups [109, 110]. Circulating miR-21, miR-34, miR-126, and miR-195 have been found to modulate the NF-κB signaling pathway in aging, inflammation, and breast cancer [111]. These miRNAs have also been found in studies of CMTs [21, 70, 76, 79–81]. miRNA levels can be also regulated by exogenous and endogenous factors, including hormones. In fact, miRNAs vary considerably with fluctuations in hormone levels throughout the menstrual cycle [112]. Estrogen can regulate miRNA expression, and the decrease in estradiol levels in postmenopausal women has been implicated in miRNA expression and age-related diseases [113]. Dogs do not experience menopause, implying an absence of reproductive senescence [114, 115]. Although fertility decreases with age, female dogs can potentially become pregnant throughout their lives. Indeed, the interval between estrus cycles increase in older female dogs [114]. However, it is unclear whether hormone levels decrease during this phase and potentially influence miRNA expression and contribute to age-related conditions in canines.

As mentioned above, miRNAs are promising biomarkers for the diagnosis and prognosis of canine mammary disease. Due to the dysregulation of specific miRNAs in carcinogenesis, miRNA-based therapy is also one of the potential applications of miRNAs. So, this type of therapy is an open field for new treatments for mammary cancer. There are two main approaches to using miRNAs as therapeutic agents: miRNAs designed to replace or increase the quantity of beneficial miRNAs (e.g., miRNA mimics) and miRNAs that aim to block miRNAs overexpressed in disease, such as miRNA inhibitors (also called anti-miRNAs or antagomiRs) and miRNA sponges that degrade and sequester miRNAs, respectively [116, 117]. A recent study observed that miR-497 and miR-195 were significantly underexpressed in CMTs compared to normal samples (average of age 10.5 years, range 6–17 years). Furthermore, using RT-qPCR, the authors showed that miR-497 was downregulated in two canine mammary tumor cell lines (CMT1211 and CMT7364) compared to normal primary canine mammary cells. The authors, by transfection, introduced miR-497 mimic into canine mammary carcinoma cells and revealed that overexpression of miR-497 decreased cell proliferation and migration and increased apoptosis in CMT1211 and CMT7364 cells. The same protocol was performed to introduce the miR-497 inhibitor and was shown to have the contrary effects in at least one cell line. As a result, miR-497 has been proposed as a diagnostic biomarker and therapeutic target in CMTs [73]. Another work, conducted by Ren and colleagues in 2022, studied the therapeutic effects of miRNA-124. The study observed that miR-124 was decreased in 20 CMTs classified as simple carcinoma and in two CMT cell lines (CHMm and CHMp) using RT-qPCR. This study also performed transfection of miR-124 mimics and inhibitors and observed that miR-124 mimics inhibited proliferation, migration, invasion and EMT in CHMm and CHMp cells. The miR-124 inhibitor showed the opposite effect. This study concluded that miR-124 directly suppressed CDH2 expression and inhibited tumor growth and EMT, suggesting it may be a potential therapeutic target [71]. In human patients, there are miRNA-targeted therapies in clinical trials, which may be a good starting point for veterinary medicine [118].

This field of research faces some challenges, such as the identification of the miRNAs involved in CMTs and the subsequent assessment of their therapeutic potential. In addition, both the development of miRNA-based therapies as well as the evaluation of their safety and efficacy are further examples of challenging factors. A particular feature of the miRNAs is that they have multiple targets, which means that the use of miRNA-based therapies may have off-target effects and lead to unintended consequences. Nevertheless, using artificial miRNAs with high similarity to the target of interest may improve treatment by reducing the risk of off-target effects and unwanted side effects. Furthermore, targeting a gene with a single miRNA may not be sufficient to produce the desired therapeutic effect. To bridge this gap, the use of a single miRNA in association with other therapies—such as chemotherapy or radiation—or even the use of a combination of several miRNAs simultaneously could be potential approaches.

Another challenge to be considered involves the development of better delivery systems to increase the stability, specificity, efficacy, low toxicity and cost-effectiveness of the therapy [119]. Lastly, it is particularly relevant to translate research results into clinical applications and assess the safety and efficacy of miRNA-based therapies in dogs.

## Conclusions

The role of miRNAs may be critical for the development and progression of CMTs, and their dysregulated expression has made miRNAs a powerful tool as cancer biomarker. miRNAs can serve as diagnostic biomarkers to assess the type of cancer or differentiate healthy tissue from tumor tissue, as well as prognostic markers to monitor disease progression and evaluate the response to therapy. In addition, they can also be metastatic markers and targets for potential therapy. Due to the genetic similarities between dogs and humans, studies in dogs often focus on miRNAs that have already been investigated and found to be relevant in human breast cancer since miRNAs can target genes that are conserved across species. The homology between species not only makes it possible to use the dog as a model animal for translational medicine, but also to transfer established therapeutic and diagnostic approaches from dogs to humans and vice versa. In this review, we found that the studies focus on canine mammary cancer cell lines, serum and mammary tissue samples, with the RT-qPCR technique being the most commonly used by researchers. Forty-three miRNAs were observed highly dysregulated, being 19 downregulated, 18 upregulated and 6 with controversial results (up- and downregulated). The most deregulated miRNAs found are miR-21, which is overexpressed, followed by miR-29b. However, miR-29b, miR-10b, miR-125a, miR-143, miR-203 and miR-214 show controversial results in different studies. miR-125a was found differently expressed between tissue and serum samples while miR-214 showed discrepancies between serum and cell lines. Other miRNAs showed different results within the same type of samples. In some cases, this is likely due to the type of sample used and the data analysis since comparing results from tumors with metastases or tumors with normal tissue as well as between histological types and grades, may alter the observed expression levels. In fact, the correlation between different samples, such as blood and the tumor, is controversial for some miRNAs. The use of miRNAs as biomarkers in veterinary medicine has yet to become a reality. The logistics of processing the samples, the low number of samples, the high cost, and the lack of standardization of procedures are among the several limitations. Future studies are needed to analyze the expression of miRNAs at different ages and among the different molecular subtypes of CMTs and breeds, identify specific molecular targets, and develop new therapies. In summary, further research is required to fully understand the complex functions of miRNAs in the development and progression of CMTs and to develop viable miRNA-based therapies for this oncological disease.

## Data Availability

There is no original data in this review article.
